# Teachers’ mental health during the first two waves of the COVID-19 pandemic in Poland

**DOI:** 10.1371/journal.pone.0257252

**Published:** 2021-09-23

**Authors:** Tomasz Daniel Jakubowski, Magdalena Maja Sitko-Dominik

**Affiliations:** Institute of Psychology, Faculty of Social Sciences, University of Silesia in Katowice, Katowice, Poland; Aalborg University, DENMARK

## Abstract

**Background:**

Teaching work is stressful, moreover during the pandemic teachers’ stress might have been intensified by distance education as well as by limited access to social support, which functions as a buffer in experiencing stress. The aim of the research was to investigate the relation between distance education and teachers’ well-being, and their close relations and other social relations during the first two waves of the COVID-19 pandemic.

**Methods:**

The research was conducted in two stages on 285 Polish primary and secondary school teachers who were recruited by means of the chain referral method. The following measures were used: The Depression Anxiety & Stress Scales-21, Berlin Social Support Scales, The Relationship Satisfaction Scale and The Injustice Experience Questionnaire.

**Results:**

The teachers experienced at least mild levels of stress, anxiety and depression, both during the first as well as the second waves of the COVID-19 pandemic in Poland. It has been confirmed that there is a negative relation between relationship quality change and social relations quality change, and stress, anxiety and depression. The variables taken into consideration in the research have provided the explanation for the variation of stress—from 6% in the first stage of the research to 47% in the second stage; for the variation of anxiety—from 21% to 31%; and for the variation of depression—from 12% to 46%, respectively.

**Conclusions:**

The research results show that due to distance work the distinction between professional work and family life might have been blurred, and as a consequence teachers’ well-being could have been worsened. The isolation put on to stop the spreading of the virus might have contributed to changes in social relations, in close relations in particular, and at the same time negatively influenced teachers’ abilities to effectively cope with the crisis situations.

## Introduction

The teaching profession has high social prestige in Poland [1], while teachers’ work is perceived as stressful and responsible, however it is not always respected [2]. Education in Poland is conducted mainly in face-to-face contact, however due to the COVID-19 pandemic, following the regulation issued by the Minister of National Education, March 11, 2020 [3], the functioning of primary and secondary was temporarily restricted until the end of the school year 2019/2020 [4]. At that time teachers, like teachers in other countries [5], were held responsible for moving education to the virtual space [6], although only 15% of Polish teachers had any experience of distance education [7].

On September 1, 2020, both primary and secondary schools were opened and education was conducted in face-to-face contact. However, from October 24, 2020 to May 16, 2021 primary school classes 4–8 were switched to distance education. In primary school classes 1–3, distance education was conducted from November 9, 2020 to January 17, 2021, while from January 18 to February 28, 2021 education in face-to-face contact was resumed [8].

Pandemic-related experiences were mostly negative, the new situation induced uncertainty. Due the restrictions aimed at preventing the spread of the virus people started having financial problems, lost work and often had to isolate from their families [9], which made people prone to symptoms of depression and anxiety [10].

Personal problems, fears for the future and health of one’s own and one’s family, attempts to meet the expectations of one’s students, their parents and the school management, experiencing financial problems, accommodation and computer shortage, and the necessity to balance professional and family responsibilities provide the reason for conducting research into teachers’ psychological well-being during the first and the second waves of the COVID-19 pandemic in Poland.

The teaching profession is highly feminized: women constitute 64% of all teachers. In the majority of world regions, women as teachers prevail in pre-primary, primary and secondary schools; Sub-Saharan Africa is an exception, where women are underrepresented as teachers in primary and secondary schools [11]. The teaching profession is also highly feminized in the EU countries, where women constitute 72% of all teachers in primary and secondary schools [12]. As the teaching profession is highly feminized in Poland [13], and Polish culture belongs to masculine cultures [14], it has been expected that the pandemic-induced situation could have serious repercussions especially for women, because in spite of the fact that the model of dual-earner marriage gets more and more popular in Poland [15], it is still believed that household and family care routines are the domain of women [16].

### Teacher stress

Teaching work is stressful, which implicates experiencing negative emotions, such as anger, anxiety, tension, frustration and depression [17]. Moreover, teacher stress may be defined in terms of existing risk and protective factors; it appears when risk factors are not counterbalanced by protective factors [18].

In spite of the fact that stress-inducing factors are specific for every teacher [17], some stress causes tend to frequently appear: workload/time pressure [19–21] low student motivation and discipline problem [21], role ambiguity and role conflict [22], pressure to introduce changes in the curriculum [23], low salary [24, 25], relations with the principal and colleagues, and unprofessional assignments [24].

Besides, workload and student behavior are considered depression and anxiety predictors [26] higher intensification of the symptoms can be observed in women, teachers with long-time professional experience, those employed in primary schools [27], and in individuals with low emotional intelligence [28].

Stress has a negative impact on teachers’ private lives, which can cause the decrease of life satisfaction, and on their professional career, which can result in lower work commitment and lower work satisfaction [26, 29–32], and can negatively affect students’ achievements [33, 34]. Stress is positively related to exhaustion and professional burnout [30]. In addition, experienced stress has a negative impact on teachers’ health, increasing the risk of psychological and behavioral disorders [24, 29, 35].

Women more frequently experience work-family conflict than family-work conflict, in spite of the fact that they equally value the performance of family roles and professional roles [36]. Professional stress is mainly caused by time and workload pressure, while family stress results from the necessity to care for children [20]. Women more often than men experience stress due to workload [37, 38], and they have life quality lower than men, which deteriorates with age [32]. This may have them make a decision to leave the profession [39].

Experiencing stress, women more often than men seek social support [37, 40]. Lack of social support, or insufficient social support, is positively related with emotional exhaustion [41]. However, it has been indicated that too frequent talk about stress may have negative consequences in the form of the decrease of professional commitment and the sense of career [40].

Among the factors which decrease stress there are emotion-regulation ability [22], mindfulness [42, 43], self-efficacy [23, 30, 44, 45], social support provided in the workplace [21, 46, 47], work-well-being activities balance [46], exercise [19], the sense of humor [48], and prayer [49].

Support provided by the school principal and the colleagues is positively related to professional satisfaction, while support provided by the family and the principal is related to life satisfaction [50]. Self-efficacy [44, 51], emotional intelligence [52, 53] and social support in the workplace are negatively related to professional burnout [51, 53, 54], which, like stress, happens more frequently among women and teachers working in low-income schools [44].

### Teacher stress in the age of COVID-19

Stress experienced by teachers is between a medium and a high level [55, 56], which has been largely caused by the transfer of education to the virtual space [6]. However, it should be stressed that teachers’ adaptation to distance education has happened relatively fast [57]. That is why, after the initial increase of exhaustion and cynicism, the increase of the effectiveness and sense of educational activities was observed [58], while teachers noticed both advantages and disadvantages of the solution.

The problems are largely caused by lack of knowledge and skills necessary for distance education [55, 59, 60], as well as by time-consuming character of the process [7, 61], and by blurring the boundaries between private and professional lives [62]. Distance education requires the cooperation between parents, teachers and students [63], however parents are not always able to get involved in the education of their children [60], they try to contact the teachers beyond the work hours, which contributes to the increase of the teachers’ stress and frustration [7]. Polish teachers also signal such problems, as experiencing exhaustion and isolation and not being paid for working overtime [61].

The results of international research show that women more often than men experience depression and anxiety symptoms due to pandemic [10]. On the one hand, female teachers experience greater stress than male teachers, and despite employing adaptation methods of stress management [55], they often have to choose between caring for their children and helping them to do their homework, and doing their job [7, 9, 62]. On the other hand, teachers have found new possibilities in distance education [64]—the stress and costs related to commuting to work have disappeared, and they have an opportunity to better manage their time [65]. To better cope with anxiety, they spend time with family, pursue new hobbies or are active in social media [66].

The imposed isolation has created an opportunity for the families to spend more time together, and, at the same time, provide mutual support [67, 68], which helps their members to effectively manage stress [69, 70]. This unique situation might also contribute to the increase of experienced stress caused by distance education. Blurring the boundaries between family and professional lives [71], and using avoidance strategies in stress management [56] could negatively affect the individuals, and, among others, contribute to the intensification of anxiety and depression symptoms [10].

In such a situation, teachers can get limited social support. Staying together with their family at home all the time may evoke negative communicative behaviors in the dyad, resulting in the deterioration of the relationship quality [67, 72–74], lack of satisfaction from the relationship, and as a consequence lowered psychological well-being [69]. The stress related to child-rearing could be amplified by depression and anxiety symptoms and stress induced by the pandemic [75].

The aim of the research is to investigate the relation between distance education and teachers’ well-being, and their close relations and other social relations during the first and second wave of the COVID-19 pandemic.

In the first stage of the research, taking into consideration the research literature on on the topic, the following hypotheses were formulated:

1 Satisfaction from the relationship, satisfaction from social relations and perceived social support negatively predict the levels of mental distress (stress, anxiety and depressive symptomatology).

In the second stage of the research, as the research group and the area of research were extended taking into consideration the disproportionately higher levels of COVID-19 cases in the Silesian voivodeship than in other voivodeships, the following hypotheses were added:

2 The sense of injustice positively predicts the levels of mental distress (stress, anxiety and depressive symptomatology).
3 The teachers participating in the research during the first wave of the pandemic experience lower levels of mental distress (stress, anxiety and depressive symptomatology) than those participating in the research during the second wave.
4 Teachers from Silesian Voivodeship experience higher levels of mental distress (stress, anxiety and depressive symptomatology) than those from other voivodeships in Poland.

## Materials and methods

### Participants and procedure

The Ethics Committee in the University of Silesia in Katowice granted the permission for conducting the research (no. KEUS.93/02.2021). The research consisted of two stages. 285 Polish teachers working in primary and secondary schools participated in it. They were recruited by means of the chain referral method. The protocol of the current study—dx.doi.org/10.17504/protocols.io.bseknbcw [PROTOCOL DOI].

#### The first stage of the research

In order to diagnose the teachers’ psychological well-being, the retrospective research, concerning the period when the research participants were engaged in distance education (March 16—June 26, 2020), was conducted in September and October 2020. The stage was carried out in the workplace, as the primary schools in the Silesian Voivodeship were open at that time.

In the first stage of the research, the participants were recruited by means of convenience sampling: the researchers directly contacted primary school principals in the Silesian Voivodeship, strictly complying with the recommendations of the Chief Sanitary Inspector. The principals were informed about the aim of the research and saw the questionnaire for teachers. On granting the permission for conducting the research, the principals were handed the questionnaires. The research was conducted by means of the traditional paper-and-pencil method. Each person that agreed to participate in the research was asked to fill in three questionnaires and provide the sociodemographic data. The participants did not get any payment for participating in the research. 200 sets of the questionnaires were handed out, 152 were returned (76%), out of which 7 (4.6%) were excluded due to incomplete data. 145 primary school teachers participated in the research, 130 women (89.7%) and 15 men (10.3%).

#### The second stage of the research

As the schools were closed again, in the second stage of the research we decided to look for factors responsible for the teachers’ current psychological well-being. The research was conducted via the Internet, through Google forms with an appended consent form, from December 2020 to February 2021, among primary and secondary school teachers.

In the second stage of research, recruitment of participants was conducted by means of snowball sampling. In order to get a representative nationwide group, the authors, together with three students acting as research assistants, tried to directly contact teachers, or sent requests concerning the participation in the research to selected schools, as well as posted the information about the research project on Internet fora for teachers. Each person that agreed to participate in the research was asked to fill in the four questionnaires and provide the sociodemographic data. The participants did not get any payment for participating in the research. 140 sets of questionnaires were filled in. 140 teachers participated in the research: 121 women (86.4%) and 19 men (13.6%).

### Measures

In the first stage of the research, dependent variables were measured by The Depression Anxiety & Stress Scales-21 (DASS-21), while independent variables were measured by The Berlin Social Support Scales (BSSS), The Relationship Satisfaction Scale (RS-10) and sociodemographic questions. In the second stage of the research, dependent variables were measured by DASS-21, while independent variables were measured by BSSS, RS-10, The Injustice Experience Questionnaire (IEQ) and sociodemographic questions.

#### Sociodemographic data

In the first stage of the research, the following sociodemographic data were gathered—gender; age; years of work as a teacher; marital status; relationship breakup during the pandemic; total number of children, including: number of children up to 8 years old, number of children 9–15 years old, number of children 16–19 years old; partner’s work status; relationship quality change during the pandemic (between March 16, 2020 and June 27, 2020); and social relations quality change during the pandemic (between March 16, 2020 and June 27, 2020).

In the case of relationship/social relations quality change during the pandemic, the respondents had to provide an answer on a 5-point Likert scale (1—“It has definitely worsened”; 2—“It has worsened”; 3—“It has not changed”; 4—“It has improved”; 5—“It has definitely got better”).

In the second stage of the research, the following sociodemographic data were gathered—gender; age; years of work as a teacher; marital status; relationship breakup during the pandemic; total number of children, including: number of children up to 8 years old, number of children 9–15 years old, and number of children 16–19 years old; partner’s work status; relationship quality change during the pandemic; social relations quality change during the pandemic; voivodeship of residence; and level of school in which a teacher is working.

In the case of relationship/social relations quality change during the pandemic, the respondents had to provide an answer on a 5-point Likert scale (1—“It has definitely worsened”; 2—“It has worsened”; 3—“It has not changed”; 4—“It has improved”; 5—“It has definitely got better”).

**DASS-21** is a short form of a DASS-42 [76] translated into Polish by the authors of the paper, consisting of 21 items divided into three sub-scales of depression (e.g., “I didn’t experience any positive feelings at all”), anxiety (e.g., “I was worried about situations in which I might panic and make a fool of myself”) and stress (e.g., “I found it hard to wind down”), answered on a 4-point Likert scale (0—“It did not apply to me at all”; 1—“It applied to me to some degree, or some of the time”; 2—“It applied to me to a considerable degree or a good part of the time”; 3—“It applied to me very much or most of the time”). Each sum of the sub-scales’ scores indicates the level of severity of the given symptoms experienced last week. Each sub-scale has cut-off points for the symptom severity level—mild (15–18 points in the stress sub-scale, 8–9 points in the anxiety sub-scale, and 10–13 points in the depression sub-scale), moderate (19–25 points in the stress sub-scale, 10–14 points in the anxiety sub-scale, and 14–20 points in the depression sub-scale), severe (26–33 points in the stress sub-scale, 15–19 points in the anxiety sub-scale, and 21–27 points in the depression sub-scale), extremely severe (34+ points in the stress sub-scale, 20+ points in the anxiety sub-scale, and 28+ points in the depression sub-scale) [76].

The sub-scale reliability was α = .90, α = .87 and α = .89 in the first stage of the research, and α = .90, α = .90 and α = .91 in the second stage of the research, for depression, anxiety and stress, respectively. The respondents were asked to say how they felt between March 16, 2020 and June 27, 2020—in the first stage of the research, and during the second wave of the pandemic—in the second stage of the research, instead of a week prior to filling in the test.

**RS-10** by Røysamb, Vittersø, and Tambs [77], translated into Polish by the authors of the paper. The scale consists of 10 items (e.g., “I am satisfied with my relationship with my partner”) answered on a 6-point Likert scale (1 -“I strongly disagree”; 2—“I disagree”; 3—“I somewhat disagree”; 4—“I somewhat agree”; 5—“I agree”; 6—“I strongly agree”). The higher is the sum of the scores, the higher is relationship satisfaction.

The reliability of the whole scale was α = .91 in the first stage of the research, and α = .93 in the second stage of the research.

**BSSS** by Schwarzer and Schulz [78] in the Polish version by Łuszczyńska, Kowalska, Schwarzer and Schulz [79] consists of 38 items divided into six scales—perceived available support, actually received support, support seeking, need for support, protective buffering and provided support. In our study, the perceived available support scale was used with its sub-scales of emotional support (e.g., “Whenever I am sad, there are people who comfort me”) and instrumental support (e.g., “I know persons on whom I can count”).

The reliability of the whole scale was α = .90 in the first stage of the research, and α = .91 in the second stage. The sub-scale reliability was α = .79, α = .87 in the first stage of the research, and α = .80, α = .90 in the second stage, for emotional and instrumental support, respectively.

**IEQ** by Sullivan [80] in Polish translation by the authors of the paper. The questionnaire measures the perceived level of experienced injustice, and consists of 12 items divided into two sub-scales of blame/unfairness (e.g., “It all seems so unfair”) and severity/irreparability (e.g., “No one should have to live this way”), answered on a 5-point Likert scale (0—“never”; 1—“rarely”; 2—“sometimes”).

The reliability of the whole scale in the current study was α = .93; the sub-scale reliability was α = .90, α = .84 for blame/unfairness and severity/irreparability, respectively. In the current study, the items addressing the medical/health condition were replaced with those addressing the life situation to measure the perceived level of experienced injustice during the lockdown and distance teaching.

### Statistical analysis

#### The first stage of the research

The sociodemographic data which were missing in the cases included in the statistical analysis were not completed. Only the information provided by the participants was taken into consideration. The study included the stress, anxiety and depression sub-scales of DASS-21 as dependent variables; and sociodemographic data, relationship and social relations quality change during the pandemic, general social support, emotional social support, instrumental social support (measured by BSSS) and relationship satisfaction (measured by RS-10) as independent variables. The analytics software package Statistica version 13.1. [81] was used to compute descriptive statistics, correlations, intergroup differences (the Mann–Whitney U test), univariate regression and multivariate backward stepwise regression.

#### The second stage of the research

The study included the stress, anxiety and depression sub-scales of DASS-21 as dependent variables; and sociodemographic data, relationship and social relations quality change during the pandemic, general social support, emotional social support and instrumental social support (measured by BSSS), relationship satisfaction (measured by RS-10), perceived injustice, blame/unfairness and severity/irreparability (measured by IEQ) as independent variables. The analytics software package Statistica version 13.1. [81] was used to compute descriptive statistics, correlations, intergroup differences (the Mann–Whitney U test), univariate regressions and multivariate backward stepwise regressions.

The statistical significance was set on p < .05. In the second stage of the research, an analysis of intergroup differences in levels of stress, depression and anxiety was conducted with the Bonferroni correction for multiple comparisons.

## Results

### The first stage of the research

The studied group consisted of 130 female and 15 male teachers working at primary schools in the Silesian voivodeship, with the mean age of 43.76 (SD = 8.31) and average work experience of 19.07 (SD = 9.11). For further information on the sociodemographic characteristics of the respondents see S1 Table.

Taking into consideration the cut-off points for symptom severity in DASS-21, 21 participants (14.48%) declared mild, 24 (16.55%)—moderate, 9 (6.21%)—severe and 10 (6.90%)—extremely severe depressive symptomatology. 15 participants (10.34%) declared mild, 25 (17.24%)—moderate, 10 (6.90%)—severe and 18 (12.41%)—extremely severe anxiety. While 21 (14.48%) declared mild, 21 (14.48%)—moderate, 14 (9.66%)—severe and 10 (6.90%)—extremely severe stress.

The analysis of the correlation between levels of stress, anxiety, depressive symptoms and sociodemographic data, relationship and social relations quality change during the pandemic, general social support, emotional social support, instrumental social support and relationship satisfaction are presented in S2 Table.

The analysis revealed positive associations between stress and age (.198), total number of children (.270) and number of children 16–19 years old (.237) and negative associations with relationship quality change during the pandemic (-.289) and social relations quality change during the pandemic (-.220). In the case of anxiety, negative associations were revealed with relationship quality change during the pandemic (-.358), social relations quality change during the pandemic (-.179), general social support (-.204), emotional social support (-.195) and relationship satisfaction (-.276). While depression was negatively associated with number of children up to 8 years old (-.165), relationship quality change during the pandemic (-.372), social relations quality change during the pandemic (-.329), general social support (-.272), emotional social support (-.224), instrumental social support (-.267) and relationship satisfaction (-.230).

To explore potential gender differences in stress, anxiety, depression, general social support, emotional social support, instrumental social support, relationship satisfaction, relationship quality change during the pandemic, and social relations quality change during the pandemic, the Mann-Whitney U test was utilized due to the non-normal distribution of the above-mentioned variables (see S3 Table). The analysis did not reveal any statistically significant gender-related differences.

Next a series of univariate regressions were conducted (see S4 Table), with stress as a dependent variable; and gender, age, being in a relationship, relationship quality change during the pandemic, relationship breakup during the pandemic, total number of children, number of children up to 8 years old, number of children 9–15 years old, number of children 16–19 years old, partner working in a regular workplace, partner working from home, partner losing job/being unemployed, social relations quality change during the pandemic and relationship satisfaction as independent variables. The analysis revealed age (β = .24), total number of children (β = .29), number of children 9–15 years old (β = .17), number of children 16–19 years old (β = .21), and partner lost job/unemployed (β = .23) as positive predictors of level of stress, while relationship quality change during the pandemic (β = -.25) was found to be a negative predictor.

Then a series of univariate regressions was conducted, with anxiety as a dependent variable; and with gender, being in a relationship, relationship quality change during the pandemic, relationship breakup during the pandemic, number of children 9–15 years old, partner working in a regular workplace, partner working from home, partner lost job/unemployed, social relations quality change during the pandemic, general social support, emotional social support, and relationship satisfaction as independent variables. The analysis revealed that positive predictors of level of anxiety were number of children 9–15 years old (β = .18) and partner lost job/unemployed (β = .32). While negative predictors of level of anxiety were relationship quality change during the pandemic (β = -.31) and relationship satisfaction (β = -.25).

Next a series of univariate regressions was conducted, with depression as a dependent variable; and with gender, being in a relationship, relationship quality change during the pandemic, relationship breakup during the pandemic, number of children up to 8 years old, number of children 9–15 years old, partner working in a regular workplace, partner working from home, partner lost job/unemployed, social relations quality change during the pandemic, general social support, emotional social support, instrumental social support, relationship satisfaction as independent variables. The analysis revealed partner losing job/being unemployed (β = .28) as a positive predictor of level of depressive symptomatology. While relationship quality change during the pandemic (β = -.37), social relations quality change during the pandemic (β = -.24), general social support (β = -.21), emotional social support (β = -.21), instrumental social support (β = -.19) and relationship satisfaction (β = -.30) were found to be negative predictors.

Next a series of multivariate regressions was conducted, with stress, anxiety and depression as dependent variables, and their respective independent predictors as independent variables.

In the final step of stepwise backward regression, from among the variables (age, total number of children, number of children 9–15 years old, number of children 16–19 years old, partner lost job/unemployed and relationship quality change during the pandemic), only number of children 9–15 years old was included in the model accounting for 6% of stress variance (S5 Table).

From among the variables (number of children 9–15 years old, partner lost job/unemployed, relationship quality change during the pandemic and relationship satisfaction), in the final step of stepwise backward regression, number of children 9–15 years old (β = .32) and relationship satisfaction (β = -.35) were included in the model accounting for 21% of anxiety variance.

From among the variables (partner losing job/being unemployed, relationship quality change during the pandemic, social relations quality change during the pandemic, general social support, emotional social support, instrumental social support and relationship satisfaction), in the final step of stepwise backward regression, only relationship quality change during the pandemic (β = -.34) was included in the model accounting for 12% of depression variance.

### The second stage of the research

The studied group consisted of 121 female teachers and 19 male teachers working at primary and secondary schools in Poland, with mean age of 43.76 (SD = 8.31) and average work experience of 19.07 (SD = 9.11). For further information on the sociodemographic characteristics of the respondents see S1 Table.

Taking into consideration the cut-off points for symptom severity in DASS-21 [76], 23 participants (16.42%) declared mild, 22 (15.71%)—moderate, 13 (9.29%)—severe and 19 (13.57%)—extremely severe depressive symptomatology. 8 participants (5.71%) declared mild, 26 (18.58%)—moderate, 11 (7.86%)—severe and 26 (18.58%)—extremely severe anxiety. While 23 participants (16.42%) declared mild, 22 (15.72%)—moderate, 13 (9.29%)—severe and 19 (13.57%)—extremely severe stress.

The analysis of the correlation (see S2 Table) between levels of stress, anxiety and depressive symptoms; and sociodemographic data, relationship and social relations quality change during the pandemic, general social support, emotional social support, instrumental social support, relationship satisfaction, perceived injustice, blame/unfairness, severity/irreparability revealed positive associations between stress and perceived injustice (.359), blame/unfairness (.581), severity/irreparability (.630); and negative ones between stress and relationship quality change during the pandemic (-.294), social relations quality change during the pandemic (-.405), general social support (-.236), emotional social support (-.248), instrumental social support (-.198) and relationship satisfaction (-.254). In the case of anxiety, positive associations were revealed with perceived injustice (.326), blame/unfairness (.545), severity/irreparability (.566); and negative ones with relationship quality change during the pandemic (-.233), social relations quality change during the pandemic (-.405), general social support (-.230), emotional social support (-.226), instrumental social support (-.204) and relationship satisfaction (-.217). While depression was positively associated with perceived injustice (.338), blame/unfairness (.650) and severity/irreparability (.609); and negatively associated with relationship quality change during the pandemic (-.305), social relations quality change during the pandemic (-.486), general social support (-.283), emotional social support (-.273), instrumental social support (-.254) and relationship satisfaction (-.253).

To explore potential gender-related and voivodeship differences in stress, anxiety, depression, general social support, emotional social support, instrumental social support, relationship satisfaction, relationship quality change during the pandemic, social relations quality change during the pandemic, perceived injustice, blame/unfairness and severity/irreparability, the Mann-Whitney U test was utilized due to the non-normal distribution of the above-mentioned variables (see S3 and S6 Tables). To solve potential problems stemming from multiple comparisons, the Bonferroni correction was applied.

In the case of four variables, level of stress, level of depression, emotional social support and instrumental social support, statistically significant gender-related differences were observed (see S3 Table). Women declared significantly higher levels of stress (M = 16.15; SD = 11.18) and depressive symptomatology (M = 13.32; SD = 11.30) than men (M = 9.79; SD = 7.91; M = 7.16; SD = 7.98, respectively). This was also the case with emotional and instrumental social support. The differences between the respondents from the Silesian voivodeship and other voivodeships were observed in general social support, instrumental social support and social relations quality change during the pandemic. In all the above mentioned cases, the respondents from the Silesian voivodeship scored significantly higher than those from other voivodeships (see S6 Table).

First, a series of univariate regressions was conducted (see S4 Table), with stress as a dependent variable, and gender, being in a relationship, relationship quality change during the pandemic, relationship breakup during the pandemic, number of children 9–15 years old, partner working in a regular workplace, partner working from home, partner losing job/being unemployed, social relations quality change during the pandemic, voivodeship of residence, level of school, general social support, emotional social support, instrumental social support, relationship satisfaction, perceived injustice, blame/unfairness severity/irreparability as independent variables. Gender (β = .20), perceived injustice (β = .34), blame/unfairness (β = 60) and severity/irreparability (β = .63) were found to be positive predictors of stress; while relationship quality change during the pandemic (β = -.32), social relations quality change during the pandemic (β = -.44), general social support (β = -.24), emotional social support (β = -.25), instrumental social support (β = -.20), and relationship satisfaction (β = -.20)—negative predictors of stress.

Second, a series of univariate regressions was conducted, with anxiety as a dependent variable; and with gender, being in a relationship, relationship quality change during the pandemic, relationship breakup during the pandemic, number of children 9–15 years old, partner working in a regular workplace, partner working from home, partner losing job/being unemployed, social relations quality change during the pandemic, voivodeship of residence, level of school, general social support, emotional social support, instrumental social support, relationship satisfaction, perceived injustice, blame/unfairness and severity/irreparability as independent variables. In the analysis, relationship breakup during the pandemic (β = .17), perceived injustice (β = .30), blame/unfairness (β = .54) and severity/irreparability (β = .56) were found to be positive predictors of anxiety, while relationship quality change during the pandemic (β = -.27), social relations quality change during the pandemic (β = -.37), general social support (β = -.24), emotional social support (β = -.24), instrumental social support (β = -.20) and relationship satisfaction (β = -.20)—negative predictors of anxiety.

Third, a series of univariate regressions was conducted, with depression as a dependent variable, and gender, being in a relationship, relationship quality change during the pandemic, relationship breakup during the pandemic, number of children 9–15 years old, partner working in a regular workplace, partner working from home, partner losing job/being unemployed, social relations quality change during the pandemic, voivodeship of residence, level of school, general social support, emotional social support, instrumental social support, relationship satisfaction, perceived injustice, blame/unfairness and severity/irreparability as independent variables. The analysis showed that gender (β = .19), perceived injustice (β = .31), blame/unfairness (β = .65) and severity/irreparability (β = .62) are positive predictors of depression. While relationship quality change during the pandemic (β = -.38), social relations quality change during the pandemic (β = -.45), general social support (β = -.32), emotional social support (β = -.32), instrumental social support (β = -.29) and relationship satisfaction (β = -.26) are negative predictors of depression.

Next, a series of multivariate regressions was conducted, with stress, anxiety and depression as dependent variables, and with their respective independent predictors as independent variables.

From among the variables (gender, perceived injustice, blame/unfairness, severity/irreparability, relationship quality change during the pandemic, social relations quality change during the pandemic, general social support, emotional social support, instrumental social support and relationship satisfaction), in the final step of stepwise backward regression, gender (β = .14), relationship quality change during the pandemic (β = -.11), social relations quality change during the pandemic (β = -.17), blame/unfairness (β = .21) and severity/irreparability (β = .33) were included in the model accounting for 47% of stress variance (see S5 Table).

From among the variables (revealed relationship breakup during the pandemic, perceived injustice, blame/unfairness, severity/irreparability, relationship quality change during the pandemic, social relations quality change during the pandemic, general social support, emotional social support, instrumental social support and relationship satisfaction), in the final step of stepwise backward regression, severity/irreparability (β = .56) was included in the model accounting for 31% of anxiety variance.

From among the variables (gender, perceived injustice, blame/unfairness, severity/irreparability, relationship quality change during the pandemic, social relations quality change during the pandemic, general social support, emotional social support, instrumental social support and relationship satisfaction), in the final step of stepwise backward regression, relationship quality change during the pandemic (β = .24) and social relations quality change during the pandemic (β = .56) were included in the model accounting for 46% of depression variance.

### Intergroup differences between the first and second stages of the research

Potential intergroup differences were explored, between the first and second stages of the research in sociodemographic data, stress, anxiety, depression, general social support, emotional social support, instrumental social support, relationship satisfaction; the chi-square test (see S1 Table) and the Mann-Whitney U test (see S3 Table) were utilized due to the non-normal distribution of the above-mentioned variables.

As far as sociodemographic characteristics are concerned, the participants of the first stage of the research and of the second stage significantly differed in marital status, total number of children and number of children 9–15 years old. More participants were in relationship (χ^2^ = 5.03), had more children in total as well as in the age range of 9–15 in the second stage of the research than in the first stage. The reverse was observed in the case of social relations quality change during the pandemic. Other differences between the participants from the first and the second stages of the research were not statistically significant.

## Discussion

### The first stage of the research

The first stage of the research has revealed that 45.52% of the teachers from the Silesian voivodeship experienced at least mild levels of stress, 46.90%—at least mild levels of anxiety, and 44.14%—at least mild levels of depressive symptomatology. While in the second stage of the research, during the second wave of the pandemic, 47.14% of the respondents experienced at least mild levels of stress, 50.71%—at least mild levels of anxiety, and 55.00%—at least mild levels of depressive symptomatology. Similar, yet lower levels of mental health issues were reported in the studies conducted by Kabito and Wami, [82], Stachteas and Stachteas [83], Zhao et al. [84], Ozamiz-Etxebarria et al. [85], Ozamiz-Etxebarria et al. [86], and Titheradge et al. [87]. Though higher than those reported among Polish teachers before the pandemic [88]. The data show a tendency toward the decrease of well-being and the increase of mental health issues, yet the results of the statistical analysis yield no statistically significant differences in that matter between the respondents in the first stage of the research, during the first wave of the COVID-19 pandemic, and those in the second stage, during the second wave. This might possibly mean that the initial abrupt change in life and prolonged remote-teaching and the lifestyle changes associated with it had a similar impact on mental well-being.

The first stage of the research has revealed positive associations between stress and age, total number of children and number of children 16–19 years old; and negative associations with relationship quality change during the pandemic and social relations quality change during the pandemic. The relationship between stress and age might suggest that aging, which is accompanied with potential decrease in coping resources (possibly being the result of cognitive decline) and burnout [89, 90], is associated with increased levels of stress. Similar results were observed in the study conducted by Mahfoudh et al. [91], while contrary findings were reported by Kourmousi and Alexopoulos [92]. Yet another possible explanation of this might lie in the urgent necessity for quick adjustment to the reality of remote teaching [55, 93–95], increased workload [89, 96–101] and the need to employ technology more extensively than before [102, 103], which in turn might have been another stressor. This explanation seems to be supported by the data obtained in the second stage of the research—where such a relation was not observed, which in turn might be the result of getting more time to adjust to remote teaching and having more experience in the use of computer technology [102]. Interestingly, Ssenyonga and Hecker [104] did not find any associations between sociodemographic data and the levels of stress. These differences might be the result either of sociodemographic heterogeneity of the respondents in the current study or of relative homogeneity of the sample in the above-mentioned study.

The association observed between levels of stress and total number of children may result from adherence to traditional gender role norms, according to which women get involved more in family life than in professional career [105]. In addition, persons who are in a relation and have more children tend to put family life above professional career, while mere possessing children makes women ready to sacrifice a lot for their family, which is socially expected [106]; this may contribute to experiencing stress resulting from having problems with maintaining work-family balance. The pandemic situation might have additionally increased stress, especially in women who take care of children most of the time, and worsened their well-being, in comparison to women who were ready to equally share the duties with their partners [107].

The relation between stress and having children at the age of 16–19 might be interpreted in the following way: with an increased number of children at this age range, the resources that could be used to cope with stress are “spent” on raising the children, and possibly on copying with the potential crises of their emerging adulthood as well as with the change in their situation due to the pandemic [108]. However, the results of the second stage of the research, in which the relation was found insignificant, might suggest that with time the children adjusted to remote learning and/or staying at home, which possibly lowered their level of stress which in turn decreased the tendency to get into conflict and/or the amount of resources spent on additional childrearing by their caretakers, thus nullifying the significance of this association. Positive change in quality of relationship and in other social relations during the pandemic is significantly related to lower levels of stress; this might be related to a more positive sense of self-worth as well as potential increase in social support.

In the case of anxiety, negative associations have been revealed with relationship quality change during the pandemic, social relations quality change during the pandemic, general social support, emotional social support and relationship satisfaction. The betterment of the relationship and social relations, perceived quality and satisfaction as well as social support, especially that of more emotional character, might be connected to increased resources in terms of emotion regulation [109–113], a more positive self-concept and a sense of stability [114], and thus associated with decreased levels of anxiety.

In the case of depression, negative associations were observed with number of children up to 8 years old, relationship quality change during the pandemic, social relations quality change during the pandemic, general social support, emotional social support, instrumental social support and relationship satisfaction. The observed associations might imply that with greater social support, a satisfying relationship and satisfying social relations with others, people are able to build a more positive self-concept, view others in a positive way and have a more optimistic view of ones’ own future [114]. Interestingly, in the case of number of children, only the association with the number of children up to 8 years old was significant. Contrary to our hypothesis that the relation in this case would be reverse (i.e. that increased demands of taking care of younger children might increase the burden of everyday routine and be related to increased depressive symptomatology), the results imply that younger children might, in fact, be less conflict-generating than older children and bring about increased child-rearing satisfaction and a sense of competence [114–116], which in turn might decrease the maladaptative beliefs affecting symptoms of depression among teachers from the Silesian voivodeship during the first wave of the COVID-19 pandemic.

The second stage of the research has shown positive associations between stress and perceived injustice, blame/unfairness and severity/irreparability; and negative associations between stress and relationship quality change during the pandemic, social relations quality change during the pandemic, general social support, emotional social support, instrumental social support and relationship satisfaction. The relationship between a sense of injustice and stress might suggest that perceived injustice is a product of events which together with straining the copying resources could affect the levels of stress; associations between perceived injustice and stress were found, among others, by Syed et al. [117]. Moreover, experiencing unfairness positively predicts experiencing negative affect [118]. In the case of the relation quality change during the pandemic and social relations quality change during the pandemic, an explanation similar to the one in the first stage of the research may be provided. In this study, overall relationship satisfaction as well as social support proved to be important. This might mean that the further into the pandemic overall satisfaction from the relationship, and social support in general, is getting more important as more external resources supplant more internal ones in copying with stress, in this way affecting its levels.

In the case of anxiety, positive associations have been revealed with perceived injustice, blame/unfairness and severity/irreparability; and negative ones—with relationship quality change during the pandemic, social relations quality change during the pandemic, general social support, emotional social support, instrumental social support and relationship satisfaction. In the case of the relation with perceived injustice, this might mean that the sense of injustice increases a tendency for worrying [119–121] as a problem solving or brooding strategy during situations which one may believe she/he has no control over, and in this way reinforcing the conviction of one’s low competence [114], which in turn might increase the levels of anxiety [111]. This indicates that justice sensitivity is related to anxiety symptoms [122]. In the case of social relations and support, a rationale similar to the one employed in the first stage of the research can be applied.

Depression was positively associated with perceived injustice, blame/unfairness and severity/irreparability; and negatively associated with relationship quality change during the pandemic, social relations quality change during the pandemic, general social support, emotional social support, instrumental social support and relationship satisfaction. The relation between depression and a sense of injustice might suggest that perceived injustice may result in ruminating, which in turn is associated with a decreased sense of self-worth [114] and increased depressive symptomatology [109–113, 123–125]. In the case of social relations and support, a rationale similar to the one employed in the second stage of the research can be applied.

In the first stage of the research, the analysis has not shown any statistically significant gender-related differences in stress, anxiety, depression, general social support, emotional social support, instrumental social support, relationship satisfaction, relationship quality change during the pandemic and social relations quality change during the pandemic. Interestingly, in the second stage of the research, statistically significant gender-related differences were observed–in the case of level of stress, level of depression, emotional social support and instrumental social support. The female respondents declared significantly higher levels of stress and depressive symptomatology than the male respondents—similar results were presented in Tai et al. [126]. This was also the case with emotional and instrumental social support. This might suggest that female teachers’ professional and housework burden is bigger [127] than that of male teachers. The tendency is not exclusively limited to Poland, as after summing up the time devoted to paid work and unpaid work, it turns out that women weekly work more than men [15]. In addition, it has been observed that during the lockdowns in dual-earner relationships it was women who devoted more time for unpaid work than men; and because of that they usually experienced a lower level of the work-life balance than before the pandemic [128]. As a consequence, female teachers experience higher levels of stress and depressive symptomatology than male teachers. Taking into consideration the results of the first stage of the research, we can infer that the burden has increased in the course of the pandemic in the case of female teachers and probably decreased in the case of male teachers. The gender differences observed in emotional and instrumental support might be the result of the increased skill of acquiring support in women and/or conforming to the male role norms in men, who perceive seeking social support as “unmanly”, thus those who might more strictly adhere to the norms may not be actively seeking social support. The results of the first stage of the research; imply that more traditional work/gender-related norms have been reintroduced or reinforced in some households during the pandemic [129–132], because the belief that the main women’s role is to do household chores and care for the family functions as a cultural norm [16, 133], and because, in spite of the fact that they are professionally active, women often hold traditional views on their role in the family, which may make them treat their household duties and caring for the family as an obligation [16, 134].

#### Voivodeship residence

The differences between the respondents from the Silesian voivodeship and other voivodeships have been observed in general social support (g = .448), instrumental social support (g = .460) and social relations quality change during the pandemic. In all the above mentioned cases–the respondents from the Silesian voivodeship scored significantly higher than those from other voivodeships. The differences might be explained by the specificity of Silesian culture, in which such values as community and family are highly cherished; this may have an impact on the way Silesians perceive, accept and offer social support [135].

#### Stress

In the first stage of the research, a series of univariate regressions revealed age, total number of children, number of children 9–15 years old, number of children 16–19 years old, partner losing job/being unemployed as positive independent predictors of stress; while relationship quality change during the pandemic was found to be a negative stress predictor. This may indicate that the number of children, both their total number and especially the number of those in the 9–19 age range, increases levels of stress (which might be the result of the childrens’ own struggle for adjustment [108], which coincides with the age cap of obligatory schooling, and during the pandemic—with remote schooling, which might strain both mental (e.g. in negative self-appraisals [114]) and material resources of their teacher-parents, and as a consequence increase the intensity of stress. In the case of the partner’s job loss/unemployed status, the resulting financial burden and potential marital conflicts might increase levels of stress. In the case of potential protective factors against stress among teachers during the first wave of the COVID-19 pandemic, in the first stage of the research, the results suggest that potential betterment of relationship (which could have been the result of initial increase in the time spend together) might have been predictive of lower levels of stress. Yet in the final step of the stepwise backward regression, only number of children 9–15 years old was included in the model, accounting for 6% of stress variance. From among the above-mentioned factors affecting the level of stress among teachers from the Silesian voivodeship, only the number of children in the 9–15 age range was included in the model. This could suggest that the increased time of child-rearing, children and copying with their adjustment to remote schooling resulted in higher levels of the perceived stress [108, 109, 121].

#### Anxiety

The analysis has also revealed that positive independent predictors of anxiety are number of children 9–15 years old and partner losing job/being unemployed. While the negative independent predictors of anxiety were relationship quality change during the pandemic and relationship satisfaction. The relationship between the number of children in the 9–15 age range and anxiety might mean that the potential amount of time and other resources might put a strain on the teacher parent as a consequence the coping resources are diminished in other potentially stressful situations. This in turn might pose a risk of elevated levels of stress. Another possible explanation of this relationship might lie in worrying [111, 119–121, 136] as a problem solving strategy and burnout [89, 90], applied to potential problems concerning children adjustment to the pandemic situation. A similar explanation might be provided in the case when the partner loses job or is unemployed. Among teachers from the Silesian voivodeship during the first wave of the COVID-19 pandemic, the independent protective factors of anxiety were relationship quality change during the pandemic and relationship satisfaction; they increased the level of stability, predictability of future (at least in the relationship aspect of life), which in turn affected the sense of security, and as a consequence decreased tendency to catastrophize [121] and levels of anxiety. Yet in the final step of the stepwise backward regression, only number of children 9–15 years old and relationship satisfaction were included in the model, accounting for 21% of anxiety variance. The results seem to suggest that the more immediate change in the relationship quality in the case of levels of anxiety was less predictive than the overall relationship satisfaction (and associated with it the sense of self-worth [114]) over a longer timespan in the case of the decrease of anxiety, and potential worries concerning the relation and the well-being of primary school children and potential worries being the sole bread earner in the household in the case of increase of anxiety [115, 116].

#### Depressive symptomatology

The analysis has shown that partner losing job/being unemployed positively predicts the level of depressive symptomatology, while relationship quality change during the pandemic, social relations quality change during the pandemic, general social support, emotional social support, instrumental social support, relationship satisfaction have been found as negative independent predictors. The loss of job by the partner/his or her unemployed status may increase the sense of hopelessness/reinforce maladaptative negative beliefs about one’s future [114], while social support, relationship satisfaction and betterment of relationship and social relations quality during the pandemic might possibly reinforce more adaptative beliefs about oneself, others and one’s future, and as a consequence decrease the levels of depressive symptomatology [114]. Yet in the final step of the stepwise backward regression, only relationship quality change during the pandemic was included in the model, accounting for 12% of depression variance. This might mean that, during the first wave of the COVID-19 pandemic, the immediate change in the relationship was more predictive of the decrease in the levels of the depressive symptomatology than the above-mentioned.

### The second stage of the research

#### Stress

In the second stage of the research, a series of univariate regressions has found gender, perceived injustice, blame/unfairness and severity/irreparability as positive predictors of stress, while relationship quality change during the pandemic, social relations quality change during the pandemic, general social support, emotional social support, instrumental social support and relationship satisfaction as negative predictors of stress. The results seem to suggest that, during the second wave of the COVID-19 pandemic, female teachers might be at higher risk of experiencing stress more intensely than male teachers. This could result from gender-specific socialization and traditional female gender norms reinforced (in the Polish society, where women have disproportionately more responsibilities than men [127]). That is why, in spite of being professionally active, women do the majority of household chores [137]. Besides, the number of their duties could have been increased due to the pandemic [138]. Such a relationship was not observed in the first stage of the research. This can be explained in two ways. The first explanation may be: either the coping resources were gradually diminishing overtime and the burden of this disproportion became significant; or in a response to wives’ work fatigue, men might have assumed some household duties [139], and as a consequence women were able to more effectively cope with experienced stress related to maintaining work-family balance. The second explanation may involve reverting to the more traditional/conservative ways of life during the period of prolonged crisis situation/hardships. The possible impact of the sense of injustice on the increase in levels of stress may result from brooding over perceived injustice or potential difficulties resulting from these experiences, which in consequence may increase levels of stress. While positive perception of relations in general and perceived social support, according to the results, seem to strengthen coping skills/resources, and at the same time decrease the levels of stress. However, in the final step of the stepwise backward regression, gender, relationship quality change during the pandemic, social relations quality change during the pandemic, blame/unfairness and severity/irreparability were included in the model, accounting for 47% of stress variance. The results seem to imply that gender (and possibly the gender norms and expectations, mentioned before), the severity of perceived injustice, intensity of blaming others for life situation (in this way reinforcing maladaptative beliefs about others [114]) and more immediate change in relationship and other social relations quality seem to be more predictive than the ones mentioned before in the case of the increase and decrease in levels of stress among the teachers during the second wave of the COVID-19 pandemic, respectively.

#### Anxiety

The univariate analysis has shown that identified relationship breakup during the pandemic, perceived injustice, blame/unfairness and severity/irreparability are positive predictors of anxiety; and relationship quality change during the pandemic, social relations quality change during the pandemic, general social support, emotional social support, instrumental social support and relationship satisfaction are negative predictors of anxiety. The sense of injustice might reinforce beliefs of incompetence, increased vulnerability, one’s pessimistic/hopeless future, as well as maladaptative beliefs about others, and the world in general, as dangerous and potentially harmful in interaction [114], which in turn might lead to such cognitive distortions as catastrophizing, while the potential sense of incompetence/vulnerability might lead to an increased tendency to worry and ruminate [121, 123–125] as problem solving strategies, in this way increasing levels of anxiety [109–111]. A similar explanation might be applied to the role of relationship breakup during the pandemic. While social support, overall relationship satisfaction and more immediate betterment of relationship as well as social relations during the second wave of the COVID-19 pandemic might decrease the impact of the above-mentioned maladaptative beliefs and strategies, at the same time decreasing the levels of anxiety among the teachers in the second stage of the research. Interestingly, in the final step of the stepwise backward regression, only severity/irreparability was included in the model, accounting for 31% of anxiety variance. This result might imply that the sense of hopelessness about the change of unfair life situation [114], possibly reinforced by prolonged and/or frequent experiences of perceived injustice seems to be the most predictive of increased levels of anxiety among the teachers during the second wave of the COVID-19 pandemic.

#### Depressive symptomatology

The analysis has revealed that gender, perceived injustice, blame/unfairness and severity/irreparability are positive predictors of depression, while relationship quality change during the pandemic, social relations quality change during the pandemic, general social support, emotional social support, instrumental social support and relationship satisfaction are negative predictors of depression. The potential explanation of the impact of gender (i.e. female gender) on the levels of depressive symptomatology might be similar to the one suggested in the case of the relationship between gender and anxiety—similar results were reported by Stachteas and Stachteas [83]. While the sense of injustice may be associated with reinforcement of maladaptative beliefs about self, one’s future and others, and a tendency to utilize rumination as a strategy of problem-solving [109–111, 113, 121, 123–125] which in turn might have increased the levels of depressive symptomatology among teachers during the second wave of COVID-19 pandemic. As has been mentioned in the case of anxiety, the perception of the relationship as well as other social relations and perceived social support might, on the one hand, reformulate maladaptative beliefs affecting the levels of depressive symptomatology, on the other, offer additional resources in the process of emotion regulation [109, 110, 112, 121] and problem-solving [113]. In the final step of the stepwise backward regression, relationship quality change during the pandemic and social relations quality change during the pandemic were included in the model, accounting for 46% of depression variance.

#### Intergroup differences

The analysis of intergroup differences in sociodemographic, social and mental health- related variables yielded no statistically significant results except for marital status, number of children and number of children 9–15 years old. More respondents were in relationship, had more children in total as well as in the age range of 9–15 in the second stage of the research than in the first stage of the research. The reverse was observed in the case of social relations quality change during the pandemic. The sociodemographic differences and their potential impact on the results presented in the study will be further discussed in the limitations section.

Taking into consideration our research question and formulated hypotheses, the obtained data seems to confirm Hypothesis 1 and Hypothesis 2, and deny Hypothesis 3 and Hypothesis 4.

## Conclusions and limitations of the study

Before conclusions are formulated, several limitations of the current study should be discussed. First, the first stage of the research was a retrospective study, while the second stage of the research focused on the respondents’ current mental state. This difference in the study design might have had an impact on the interstudy differences analysis–the respondents in the study pertaining to the first wave of the COVID-19 pandemic could have had problems with recalling their mental state in a specific period of the lockdown–thus the gathered data might have been constrained by the recall bias. Second, the chain referral method might have had a potentially negative impact on the data obtained from the respondents who were eager to participate and reveal information about their mental state; in this way the elicited data might have been the subject of the respondent and report bias. Third, more respondents were in a relationship, had more children in total as well as in the age range of 9–15 in the second stage of the research than in the first stage. The reverse tendency has been observed in social relations quality change during the pandemic. These differences might nullify potential intergroup differences in social support, sense of injustice and mental health condition. Besides, in the group of teachers participating in the research women prevailed (88%), which resulted from the fact that the teaching profession is highly feminized in Poland [13]. Thus, any generalizations about male teachers should be made with extreme caution. Another limitation is the use of translated and yet not adapted versions of DASS-21, RS-10 and IEQ and the fact that different teachers participated in the two studies. The latter might have impacted the results of the inter-wave comparisons.

On the basis of the analysis of the research results, the following conclusions have been drawn. The teachers experienced at least mild levels of stress, anxiety and depression, both during the first and the second waves of the COVID-19 pandemic in Poland. Social relations and close/marital relations had a great impact on the teachers’ well-being, and problems in these relations could have decreased the effectiveness of their coping with stress during the pandemic. The main conclusion from the study is that it is extremely important to establish social relations and maintain them on a satisfactory level. Those issues should as well be addressed in the workplace policies such as work counseling and trainings on problem solving and emotion regulation.

## Supporting information

S1 Table Characteristics of the participants of both stages of the research. (DOCX)

S2 Table The results of the analysis of the correlation between the studied variables in the first and second stages of the research. (DOCX)

S3 Table The results of the analysis of gender differences (the Mann-Whitney U test) between the first and the second stages of the research. (DOCX)

S4 Table Results of the series of univariate regressions. (DOCX)

S5 Table The summary of the series of multivariate stepwise backward regressions with stress, anxiety and depression as dependent variables, and with other independent variables in both stages of the research. (DOCX)

S6 Table The results of the analysis of differences related to the voivodeship (the Mann-Whitney U test) in the second stage of the research. (DOCX)
