# Supplementary material for: Teachers’ mental health during the first two waves of the COVID-19 pandemic in Poland
Source: PLoS One. 2021 Sep 23;16(9):e0257252. doi: 10.1371/journal.pone.0257252 (PMC8460021; doi:10.1371/journal.pone.0257252)
Supplement: S1 Table — (DOCX) [file pone.0257252.s001.docx]

**S1 Table. Characteristics of the participants of both stages of the research.**

|  | **1st wave of the COVID-19 pandemic (n= 145)** | **2nd wave of the COVID-19 pandemic (n= 140)** | **P** | **Effect size*** |
| --- | --- | --- | --- | --- |
| **Gender** | 130 Females (89.66%), 15 males (10.34%) | 121 Females (86.43%), 19 males (13.57%) | .401 | 0.71** |
| **Age** | M = 43.76; SD = 8.31 | M = 44.09; SD = 9.60 | .559 | 0.037 |
| **Years of work as a teacher** | M = 19.07; SD = 9.11 | M = 19.09; SD = 9.85 | .735 | 0.002 |
| **Marital Status** | 29 single (19.08%), 19 in an informal relationship (12.05%), 92 married (60.53%), 12 omissions (7.89%) | 13 Single (9.29%), 16 in an informal relationship (11.43%), 111 married (79.29%) | .025 | 5.03**^A^ |
| **Relationship quality change during the pandemic** | M = 3.13 ; SD = 1.01 | M = 2.88; SD = 0.83 | .142 | 0.270 |
| **Relationship breakup during the pandemic** | yes 4 (2.63%); no 118 (77.63%), 30 omissions (19.74%) | yes 4 (2.86%); no 136 (97.14%) | .576 | 0.31** |
| **Total number of children** | 43 have no children (28.29%), 41 have one child (26.97%), 47 have two children (30.92%), 12 have three children (7.89%), 2 have four children (1.32%), 7 omissions (4.61%); M = 1.40; SD = 0.95 | 114 have no children (81.43%), 22 have one child (15.71%), 4 have two children (2.86%);M = 1.54; SD = 1.25 | .042 | 0.126 |
| **Number of children up to 8 years old** | M = 0.37; SD = 0.61; | M = 0.29; SD = 0.69; | .769 | 0.123 |
| **Number of children 9-15 years old** | M = 0.20; SD = 0.49; | M = 0.39; SD = 0.65 | .002 | 0.278 |
| **Number of children 16-19 years old** | M = 0.41; SD = 0.71; | M = 0.21; SD = 0.48 | .177 | 0.330 |
| **Partner’s work status** | Working in a regular workplace 41 (26.97%); working from home 62 (40.79%); 9 lost job/unemployed (5.92%); 1 working both in a regular workplace and from home (0.66%), 9 omissions (25.66%) | Working in a regular workplace 95 (67.86%); working from home 38 (27.14%); 7 lost job/unemployed (5.00%), | .565 | 0.33**^B^ |
| **Social relations quality change during the pandemic** | M = 2.69; SD = 0.93 | M = 2.26; SD = 0.80 | <.001 | 0.496 |
| **Voivodeship** | 145 from Śląskie (100%) | Małopolskie – 32 (22.86%); Śląskie – 74 (52.86%); Łódzkie – 3 (2.14%); Lubuskie – 1 (0.71%); Lubelskie – 2 (1.43%); Zachodnio-Pomorskie – 6 (4.28%); Wielkopolskie – 3 (2.14%); Świętokrzyskie – 1 (0.71%); Warmińsko-Mazurskie – 3 (2.14%); Kujawsko-Pomorskie – 3 (2.14%); Opolskie – 1 (0.71%); Pomorskie – 3 (2.14%); Dolnośląskie – 4 (2.86%); Podkarpackie – 1 (0.71%); Podlaskie – 2 (1.43%); Mazowieckie – 1 (0.71%) | - | - |
| **Level of school** | 145 primary school teachers (100%) | Primary school 95 (67.86%), Secondary school 45 (32.14%) | - | - |
| **Stress** | M = 14.55; SD = 10.22 | M = 15.29; SD = 10.99 | .819 | 0.070 |
| **Anxiety** | M = 8.04; SD = 8.61 | M = 10.04; SD = 10.07 | .597 | 0.213 |
| **Depression** | M = 9.69; SD = 9.24 | M = 12.49; SD = 11.09 | .112 | 0.274 |
| **General social support** | M = 26.79; SD = 5.25 | M = 26.70; SD = 4.11 | .160 | 0.020 |
| **Emotional social support** | M = 13.13; SD = 2.77 | M = 13.14; SD = 2.15 | .116 | 0.004 |
| **Instrumental social support** | M = 13.66; SD = 2.71 | M = 13.65; SD = 2.20 | .352 | 0.004 |
| **Relationship satisfaction** | M = 47.48; SD = 10.94 | M = 44.34; SD = 16.44 | .070 | 0.225 |
| **Perceived injustice** | - | M = 55.16; SD = 16.12 | - | - |
| **Blame/unfairness** | - | M = 8.01; SD = 6.38 | - | - |
| **Severity/irreparability** | - | M = 9.29; SD = 5.91 | - | - |

S1 Table 1

*Cohen’s Effect size d; **Chi-square statistic; ^A^dummy-coded – 1= in a relationship, 0= single; ^B^dummy-coded – 1= employed, 0= unemployed.
