# Supplementary material for: Teachers’ mental health during the first two waves of the COVID-19 pandemic in Poland
Source: PLoS One. 2021 Sep 23;16(9):e0257252. doi: 10.1371/journal.pone.0257252 (PMC8460021; doi:10.1371/journal.pone.0257252)
Supplement: S2 Table — (DOCX) [file pone.0257252.s002.docx]

**S2 Table.** **The results of the analysis of the correlation between the studied variables in the first and second stages of the research.**

|  | **1st wave of the COVID-19 pandemic** | | | **2nd wave of the COVID-19 pandemic** | | |
| --- | --- | --- | --- | --- | --- | --- |
|  | **Stress** | **Anxiety** | **Depression** | **Stress** | **Anxiety** | **Depression** |
| **Age** | .198* | .023 | .008 | -.056 | .064 | -.004 |
| **Years of work as a teacher** | .130 | -.018 | -.036 | -.050 | .042 | -.006 |
| **Total number of children** | .270* | .055 | .100 | -.063 | -.103 | -.099 |
| **Number of children up to 8 years old** | -.146 | -.113 | -.165* | -.063 | -.101 | -.109 |
| **Number of children 9-15 years old** | .077 | .077 | .028 | -.013 | -.074 | -.068 |
| **Number of children 16-19 years old** | .237** | .054 | .118 | .041 | -.023 | .020 |
| **Relationship quality change during the pandemic** | -.289** | -.358*** | -.372*** | -.294** | -.233** | -.305*** |
| **Social relations quality change during the pandemic** | -.220** | -.179* | -.329*** | -.405*** | -.405*** | -.486*** |
| **General social support** | -.108 | -.204* | -.272*** | -.236** | -.230** | -.283*** |
| **Emotional social support** | -.090 | -.195* | -.224** | -.248** | -.226** | -.273** |
| **Instrumental social support** | -.093 | -.162 | -.267** | -.198* | -.204* | -.254** |
| **Relationship satisfaction** | -.089 | -.276*** | -.230*** | -.254** | -.217* | -.253** |
| **Perceived injustice** | - | - | - | .359*** | .326*** | .338*** |
| **Blame/unfairness** | - | - | - | .581*** | .545*** | .650*** |
| **Severity/irreparability** | - | - | - | .630*** | .566*** | .609*** |

S2 Table 1

**^A^**Spearman’s rank correlation coefficient, ^B^Pearson’s correlation coefficient, *p<.05, **p<.01, ***p<.001
