# Supplementary material for: Teachers’ mental health during the first two waves of the COVID-19 pandemic in Poland
Source: PLoS One. 2021 Sep 23;16(9):e0257252. doi: 10.1371/journal.pone.0257252 (PMC8460021; doi:10.1371/journal.pone.0257252)
Supplement: S3 Table — (DOCX) [file pone.0257252.s003.docx]

**S3 Table.** **The results of the analysis of gender differences (the Mann-Whitney U test) between the first and the second stages of the research.**

| **Variable** | **1st wave of the pandemic** | | | | | | | | **2nd wave of the pandemic** | | | | | | | |
| --- | --- | --- | --- | --- | --- | --- | --- | --- | --- | --- | --- | --- | --- | --- | --- | --- |
|  | **Females** | | **Males** | | **U** | **Z corr.** | **P** | **Effect Size*** | **Females** | | **Males** | | **U** | **Z corr.** | **P**** | **Effect Size*** |
|  | M | SD | M | SD |  |  |  |  | M | SD | M | SD |  |  |  |  |
| **Stress** | 14.90 | 10.04 | 16.80 | 11.93 | 920.50 | 0.35 | .725 | 0.186 | 16.15 | 11.18 | 9.79 | 7.91 | 777.50 | 2.27 | .023 | 0.588 |
| **Anxiety** | 8.27 | 8.32 | 12.20 | 11.13 | 795.00 | -1.17 | .241 | 0.458 | 10.68 | 10.27 | 6.00 | 7.72 | 826.00 | 1.98 | .048 | 0.469 |
| **Depression** | 9.52 | 8.71 | 12.00 | 11.51 | 975.00 | <.01 | .997 | 0.277 | 13.32 | 11.30 | 7.16 | 7.98 | 770.50 | 2.31 | .021 | 0.564 |
| **General social support** | 26.96 | 5.09 | 25.30 | 4.69 | 735.00 | 1.55 | .118 | 0.328 | 27.13 | 3.93 | 24.63 | 4.62 | 787.50 | 2.21 | .027 | 0.621 |
| **Emotional social support** | 13.26 | 2.66 | 12.00 | 2.58 | 792.50 | 1.18 | .231 | 0.475 | 13.27 | 2.09 | 12.32 | 2.38 | 892.50 | 1.59 | .113 | 0.446 |
| **Instrumental social support** | 13.70 | 2.63 | 13.30 | 2.50 | 768.00 | 1.34 | .171 | 0.153 | 13.86 | 2.10 | 12.32 | 2.40 | 730.50 | 2.62 | .009 | 0.719 |
| **Relationship satisfaction** | 47.41 | 11.19 | 46.80 | 9.40 | 929.50 | 0.29 | .770 | 0.055 | 43.48 | 17.28 | 49.79 | 7.79 | 1012.00 | -0.83 | .404 | 0.386 |
| **Relationship quality change during the pandemic** | 3.12 | 0.87 | 2.80 | 1.69 | 423.00 | 1.01 | .256 | 0.333 | 2.85 | 0.86 | 3.05 | 0.52 | 1005.00 | -1.01 | .311 | 0.243 |
| **Social relations quality change during the pandemic** | 2.66 | 0.90 | 2.90 | 0.99 | 674.00 | -1.55 | .086 | 0.264 | 2.24 | 0.82 | 2.37 | 0.68 | 1021.00 | -0.84 | .402 | 0.162 |
| **Perceived injustice** | - | - | - | - | - | - | - | - | 54.44 | 16.39 | 59.74 | 13.81 | 981.50 | -1.02 | .308 | 0.330 |
| **Blame/**  **Unfairness** | - | - | - | - | - | - | - | - | 8.21 | 6.56 | 6.68 | 5.00 | 1039.00 | 0.67 | .502 | 0.240 |
| **Severity/**  **irreparability** | - | - | - | - | - | - | - | - | 9.36 | 5.83 | 8.89 | 6.53 | 1083.00 | 0.40 | .687 | 0.079 |

S3 Table 1

*Hedge’s g; ** Bonferroni correction α= .025
