# Supplementary material for: Teachers’ mental health during the first two waves of the COVID-19 pandemic in Poland
Source: PLoS One. 2021 Sep 23;16(9):e0257252. doi: 10.1371/journal.pone.0257252 (PMC8460021; doi:10.1371/journal.pone.0257252)
Supplement: S4 Table — (DOCX) [file pone.0257252.s004.docx]

**S4 Table. Results of the series of univariate regressions.**

|  | **1st wave of the COVID-19 pandemic** | | | | | | | | | | | | **2nd wave of the COVID-19 pandemic** | | | | | | | | | | | |
| --- | --- | --- | --- | --- | --- | --- | --- | --- | --- | --- | --- | --- | --- | --- | --- | --- | --- | --- | --- | --- | --- | --- | --- | --- |
|  | **Stress** | | | | **Anxiety** | | | | **Depression** | | | | **Stress** | | | | **Anxiety** | | | | **Depression** | | | |
|  | Β | T | F | R2 | β | T | F | R2 | Β | T | F | R2 | Β | T | F | R2 | β | T | F | R2 | β | T | F | R2 |
| **Gender^A^** | .03 | 0.32 | 0.10 | <.01 | -.08 | -0.93 | 0.87 | .01 | <-.001 | 4.24 | <.01 | <.01 | .20* | 2.38 | 5.68 | .04 | .16 | 1.90 | 3.61 | .03 | .19* | 2.29 | 5.23 | .04 |
| **Age** | .24** | 2.89 | 8.33 | .06 |  |  |  |  |  |  |  |  |  |  |  |  |  |  |  |  |  |  |  |  |
| **Years of work as a teacher** |  |  |  |  |  |  |  |  |  |  |  |  |  |  |  |  |  |  |  |  |  |  |  |  |
| **Being in a relationship^B^** | <.01 | 0.05 | <.01 | <.01 | -.06 | -0.66 | 0.43 | <.01 | -.10 | -1.21 | 1.45 | .01 | -.06 | -0.67 | 0.45 | <.01 | -.11 | -1.26 | 1.59 | .01 | -.12 | -1.36 | 1.85 | .01 |
| **Relationship quality change during the pandemic** | -.25** | -2.75 | 7.54 | .06 | -.31*** | -3.51 | 12.31 | .10 | -.37*** | -4.22 | 17.18 | .14 | -.32*** | -4.03 | 16.28 | .11 | -.27** | -3.31 | 10.93 | .07 | -.38*** | -4.82 | 23.24 | .14 |
| **Relationship breakup during the pandemic^C^** | -.09 | -0.98 | 0.96 | .01 | -.09 | -0.95 | 0.90 | .01 | -.03 | -0.36 | 0.13 | <.01 | .15 | 1.81 | 3.27 | .02 | .17* | 2.03 | 4.12 | .03 | .21* | 2.52 | 6.35 | .04 |
| **Total number of children** | .29*** | 3.57 | 12.76 | .08 |  |  |  |  |  |  |  |  |  |  |  |  |  |  |  |  |  |  |  |  |
| **Number of children up to 8 years old** |  |  |  |  |  |  |  |  | -.12 | -1.47 | 2.17 | .02 |  |  |  |  |  |  |  |  |  |  |  |  |
| **Number of children 9-15 years old** | .17* | 2.04 | 4.17 | .03 | .18* | 2.22 | 4.92 | .03 | .14 | 1.64 | 2.68 | .02 | -.04 | -0.50 | 0.25 | <.01 | -.10 | -1.19 | 1.42 | .01 | -.11 | -1.30 | 1.69 | .01 |
| **Number of children 16-19 years old** | .21* | 2.59 | 6.70 | .05 |  |  |  |  |  |  |  |  |  |  |  |  |  |  |  |  |  |  |  |  |
| **Partner working in a regular workplace^D^** | -.09 | -0.93 | 0.86 | .01 | -.11 | -1.14 | 1.31 | .01 | -.16 | -1.74 | 3.01 | .03 | -.02 | -0.26 | 0.07 | <.01 | -.05 | -0.58 | 0.33 | <.01 | <.01 | 0.03 | <.01 | <.01 |
| **Partner working from home^E^** | -.03 | -0.36 | 0.13 | <.01 | -.05 | -0.57 | 0.33 | <.01 | .02 | 0.20 | 0.04 | <.01 | .02 | 0.25 | 0.06 | <.01 | .01 | 0.12 | 0.01 | <.01 | -.03 | -0.38 | 0.14 | <.01 |
| **Partner losing job/being unemployed^F^** | .23* | 2.53 | 6.41 | .05 | .32*** | 3.56 | 12.65 | .10 | .28** | 3.04 | 9.22 | 0.08 | .02 | 0.24 | 0.06 | <.01 | .06 | 0.73 | 0.54 | <.01 | .06 | 0.72 | 0.51 | <.01 |
| **Social relations quality change during the pandemic** | -.16 | -1.93 | 3.73 | .03 | -.07 | -0.87 | 0.75 | .01 | -.24** | -2.92 | 8.55 | .06 | -.44*** | -5.74 | 33.00 | .19 | -.37*** | -4.65 | 21.62 | .14 | -.45*** | -5.93 | 35.23 | .20 |
| **Voivodeship^G^** |  |  |  |  |  |  |  |  |  |  |  |  | <.01 | 0.05 | <.01 | <.01 | <.01 | -.02 | <.01 | <.01 | -.08 | -0.89 | 0.80 | .01 |
| **Level of school^H^** |  |  |  |  |  |  |  |  |  |  |  |  | .10 | 1.15 | 1.33 | .01 | .11 | 1.25 | 1.57 | .01 | .13 | 1.55 | 2.40 | .02 |
| **General social support** |  |  |  |  | -.10 | -1.22 | 1.50 | .01 | -.21* | -2.61 | 6.80 | .05 | -.24** | -2.89 | 8.32 | .06 | -.24** | -2.85 | 8.12 | .06 | -.32*** | -3.94 | 15.53 | .10 |
| **Emotional social support** |  |  |  |  | -.10 | -1.22 | 1.49 | .01 | -.21** | -2.63 | 6.90 | .05 | -.25** | -3.10 | 9.59 | .06 | -.24** | -2.91 | 8.49 | .06 | -.32*** | -3.90 | 15.23 | .10 |
| **Instrumental social support** |  |  |  |  |  |  |  |  | -.19* | -2.35 | 5.51 | .04 | -.20* | -2.35 | 5.52 | .04 | -.20* | -2.46 | 6.04 | .04 | -.29*** | -3.50 | 12.26 | .08 |
| **Relationship satisfaction** |  |  |  |  | -.25** | -3.06 | 9.37 | .06 | -.30*** | -3.71 | 13.77 | .09 | -.20* | -2.44 | 5.95 | .04 | -.20* | -2.34 | 5.49 | .04 | -.26** | -3.16 | 9.99 | .07 |
| **Perceived injustice** |  |  |  |  |  |  |  |  |  |  |  |  | .34*** | 4.21 | 17.72 | .11 | .30*** | 3.67 | 13.45 | .09 | .31*** | 3.81 | 14.49 | .10 |
| **Blame/unfairness** |  |  |  |  |  |  |  |  |  |  |  |  | .60*** | 8.84 | 78.20 | .36 | .54*** | 7.63 | 58.27 | .30 | .65*** | 9.96 | 99.29 | .42 |
| **Severity/irreparability** |  |  |  |  |  |  |  |  |  |  |  |  | .63*** | 9.58 | 91.71 | .40 | .56*** | 7.91 | 62.51 | .31 | .62*** | 9.21 | 84.82 | .38 |

S4 Table 1

^A^dummy-coded – 1= female, 0= male; ^B^dummy-coded – 1= in a relationship, 0= single;  ^C^dummy-coded – 1= breakup, 0= no breakup; ^D^dummy-coded – 1= working in a regular workplace, 0= not working in a regular workplace; ^E^dummy-coded – 1= partner workin from home, 0= partner not working from home; ^F^dummy-coded – 1= partner lost job/unemployed, 0= partner employed; ^G^dummy-coded – 1= Silesian voivodship, 0= other voivodship ;  ^H^dummy-coded – 1= primary school, 0= secondary school; *p<.05, **p<.01, ***p<.001
