# Supplementary material for: Teachers’ mental health during the first two waves of the COVID-19 pandemic in Poland
Source: PLoS One. 2021 Sep 23;16(9):e0257252. doi: 10.1371/journal.pone.0257252 (PMC8460021; doi:10.1371/journal.pone.0257252)
Supplement: S5 Table — (DOCX) [file pone.0257252.s005.docx]

**S5 Table. The summary of the series of multivariate stepwise backward regressions with stress, anxiety and depression as dependent variables, and with other independent variables in both stages of the research.**

| **Dependent variables** | **Independent variables** | **1st wave of COVID-19 pandemic** | | | | **2nd wave of COVID-19 pandemic** | | | |
| --- | --- | --- | --- | --- | --- | --- | --- | --- | --- |
|  |  | Β | T | F | Adj. R2 | β | T | F | Adj. R2 |
| **Stress** | Number of children between 9-15 years old | .26* | 2.81 | 7.92 | .06 | - | - | - | - |
|  | Gender^A^ | - | - | - | - | .14*** | 6.43 | 180.96 | .47 |
|  | Relationship quality change during the pandemic | - | - | - | - | -.11*** | -4.63 |  |  |
|  | Social relations quality change during the pandemic | - | - | - | - | -.17*** | -7.14 |  |  |
|  | Blame/unfairness | - | - | - | - | .21*** | 6.29 |  |  |
|  | Severity/irreparability | - | - | - | - | .33*** | 9.85 |  |  |
| **Anxiety** | Number of children between 9-15 years old | .32*** | 3.73 | 15.79 | .21 | - | - | - | - |
|  | Relationship satisfaction | -.35*** | -4.14 |  |  | - | - | - | - |
|  | Severity/irreparability | - | - | - | - | .56*** | 7.91 | 62.51 | .31 |
| **Depression** | Relationship quality change during the pandemic | -.34*** | -4.04 | 16.34 | .12 | -.24*** | -3.66 | 60.80 | .46 |
|  | Social relations quality change during the pandemic | - | - | - | - | .56*** | 8.31 |  |  |

S5 Table 1

*p<.05, **p<.01, ***p<.001 ; ^A^dummy-coded – 1= female, 0= male
