# Supplementary material for: Teachers’ mental health during the first two waves of the COVID-19 pandemic in Poland
Source: PLoS One. 2021 Sep 23;16(9):e0257252. doi: 10.1371/journal.pone.0257252 (PMC8460021; doi:10.1371/journal.pone.0257252)
Supplement: S6 Table — (DOCX) [file pone.0257252.s006.docx]

**S6 Table. The results of the analysis of differences related to the voivodeship (the Mann-Whitney U test) in the second stage of the research.**

| **Variable** | **Silesian Voivodeship** | | **Other Voivodeships** | | **U** | **Z corr.** | **P**** | **Effect Size*** |
| --- | --- | --- | --- | --- | --- | --- | --- | --- |
|  | M | SD | M | SD |  |  |  |  |
| **Stress** | 15.33 | 11.16 | 15.23 | 10.88 | 2433.50 | -0.01 | .988 | 0.009 |
| **Anxiety** | 10.03 | 10.21 | 10.06 | 9.99 | 2379.50 | -0.24 | .809 | 0.003 |
| **Depression** | 11.71 | 11.15 | 13.38 | 11.04 | 2187.00 | -1.05 | .294 | 0.150 |
| **General social support** | 27.63 | 3.64 | 25.83 | 4.42 | 1840.00 | 2.51 | .012 | 0.448 |
| **Emotional social support** | 13.52 | 2.01 | 12.71 | 2.24 | 1920.50 | 2.19 | .028 | 0.382 |
| **Instrumental social support** | 14.11 | 1.90 | 13.12 | 2.41 | 1875.50 | 2.42 | .016 | 0.460 |
| **Relationship satisfaction** | 43.89 | 18.06 | 44.85 | 14.46 | 2406.50 | 0.13 | .898 | 0.058 |
| **Relationship quality change during the pandemic** | 2.95 | 0.68 | 2.80 | 0.97 | 2302.00 | 0.65 | .514 | 0.181 |
| **Social relations quality change during the pandemic** | 2.40 | 0.82 | 2.09 | 0.74 | 1900.00 | 2.41 | .016 | 0.395 |
| **Perceived injustice** | 53.72 | 19.09 | 56.82 | 11.73 | 2348.50 | -0.37 | .711 | 0.193 |
| **Blame/Unfairness** | 7.64 | 6.08 | 8.43 | 6.73 | 2308.00 | -0.54 | .589 | 0.124 |
| **Severity/irreparability** | 9.23 | 6.03 | 9.37 | 5.82 | 2379.50 | -0.24 | .810 | 0.024 |

S6 Table 1

*Hedge’s g** Bonferroni correction α= .025
